# Exploring Representations of Old Age Emerging from the Narratives of Healthcare Professionals Working with Older Adults in Two Italian Cities

**DOI:** 10.3390/bs16091620

**Published:** 2026-09-10

**Authors:** Barbara Cordella, Michela Di Trani, Alessia Renzi, Maria Gattuso, Ambra Galiccia, Paola Elia, Maria Giovanna Massari, Andrea Greco, Francesca Morganti

**Affiliations:** 1 Department of Dynamic, Clinical Psychology and Health Studies, Sapienza University of Rome, Via degli Apuli, 1, 00185 Rome, Italy; barbara.cordella@uniroma1.it (B.C.); michela.ditrani@uniroma1.it (M.D.T.); elia.paol@tiscali.it (P.E.); 2 Department of Human and Social Sciences, University of Bergamo, Piazzale S. Agostino, 2, 24129 Bergamo, Italy; maria.gattuso@unibg.it (M.G.); ambra.galiccia@unibg.it (A.G.); andrea.greco@unibg.it (A.G.); francesca.morganti@unibg.it (F.M.); 3 Department of Human Sciences and Promotion of Quality of Life, San Raffaele Telematic University, Via di Val Cannuta, 247, 00166 Rome, Italy; giovanna.massari@uniroma5.it

**Keywords:** aging, healthcare professionals, social representations, professional discourse, cultural meanings, Emotional Text Mining, ageism

## Abstract

Population ageing is reshaping care systems and cultural meanings of later life. Present explorative qualitative study examined how healthcare professionals working with older adults in two Italian cities (Rome and Bergamo), represent old age. Moreover, we explored whether these representations vary by age, gender, and location. Fifty professionals aged 30–60 years, each residing and practicing professionally in either Rome (*n* = 25) or Bergamo (*n* = 25), were interviewed using an open-ended prompt on resources, difficulties, and transitions related to old age. A quali-quantitative approach based on Emotional Text Mining (ETM) was applied to verbatim transcripts. The analysis included text preprocessing, bisecting k-means clustering, and correspondence analysis. Seven clusters emerged: Witnessing ageing, Passing years, Challenging old age, Denial, Awareness, Rethinking services, and Old age as a problem. Correspondence analysis identified six factors; the first three, accounting for 60.24% of total inertia, were interpreted as Time (Present vs. Future), Places (Protected Shelter vs. Exposure to Risk), and Care (Assistance vs. Planning). Together, the factors and clusters outlined a symbolic field ranging from direct observation of ageing and reflection on time, to defensive responses and concerns about decline, and to greater awareness of older adults’ needs and calls for more responsive services. No statistically significant differences emerged by age or gender. Conversely, cluster distribution was significantly associated with participants’ location (Rome vs. Bergamo; χ^2^ = 49.84, *p* < 0.001), suggesting possible differences in the representation of older adults across these two geographical areas. Overall, representations oscillated between proximity and defense, individual responsibility and system-level delegation, supporting reflective training and organizational interventions to strengthen care cultures.

## 1. Introduction

In recent decades, population aging has become one of the most significant and transversal demographic phenomena globally: a process unprecedented in human history in terms of speed and breadth, determined by the combination of declining fertility, increasing longevity and the advancement of large cohorts towards old age, as well as advances in medicine and healthcare, which are recognized as being among the main factors in the extension of average life expectancy (United Nations, Department of Economic and Social Affairs, Population Division, 2024).

In 2015, the World Health Organization (WHO) published its first Global Report on Aging and Health, highlighting how increasing life expectancy represents a social achievement, but also a complex challenge for health, economic, and cultural systems (World Health Organization, 2015). With regard to the Italian context, the older adult population is constantly growing. Between 2002 and 2021, the share of people aged 65 or over rose from 12.4% to 23.5%, with projections estimating a further increase to around 35% by 2050 (Istituto Nazionale di Statistica, 2022). Moreover, it can be noted that almost one in three older people in Italy (32.4%) live with serious or multiple chronic diseases; more than half of them face at least three chronic conditions simultaneously (Istituto Nazionale di Statistica, 2021). All these factors contribute to the increasing need for “formal” and family care, particularly in relation to assistance with instrumental activities of daily living and the management of age-related physical and psychological needs (Santini et al., 2025).

The aging of the population is a complex phenomenon that cannot be reduced to demographic dynamics alone but is intertwined with the growing complexity of care and the transformation of needs in everyday life (Calderón-Jaramillo & Zueras, 2023; Morganti, 2024).

In this regard, caring for older people also entails addressing the interaction among three distinct systems: older people themselves, the healthcare professionals who care for them, and their formal and/or informal caregivers. Indeed, the broader project from which the present study emerged aimed to explore the perspectives through which older people, caregivers, and healthcare professionals discuss and make sense of old age.

With specific regard to the perspective of healthcare professionals, a recent qualitative systematic review examined healthcare professionals’ attitudes toward aging, highlighting the coexistence of multiple and sometimes contrasting perspectives (Jeyasingam et al., 2023). Caring for older adults was described as complex and demanding (Higashi et al., 2012), often associated with experiences of dependency and loss of autonomy (Bulut et al., 2015), but also with recognition of their resilience and relational richness (Lee & Richardson, 2020). Within this framework, the concept of “older adult” appears to be shaped less by chronological age than by levels of autonomy and the presence of frailty. Family involvement also emerged as a key factor influencing professionals’ experiences, which may range from collaboration to frustration. Finally, these representations are shaped by organizational conditions, including limited resources, compassion fatigue, and burnout, which may contribute to more defensive or emotionally detached attitudes (Jeyasingam et al., 2023).

Due to their continuous exposure to frail and vulnerable older adults, often in resource-limited contexts, healthcare professionals may be particularly at risk of developing ageist attitudes (Crutzen et al., 2022). These attitudes can negatively influence the quality of care and contribute to the internalization of ageism among older adults (Fernández-Puerta et al., 2024). Research has therefore increasingly focused on identifying the factors underlying ageist attitudes. Palsgaard et al. (2022) showed that anxiety about one’s own aging does not directly predict ageism, but may contribute to negative expectations about later life, which in turn foster ageist attitudes. Conversely, greater knowledge of aging and more positive views of later life are associated with lower levels of ageism. Moreover, it is important to underscore that healthcare professionals are not only privileged observers of aging but are themselves increasingly involved in the demographic changes affecting society. Recent reports indicate that the healthcare workforce is progressively aging across Europe, meaning that many healthcare professionals experience aging not only through their patients but also in their own lives and family contexts (World Health Organization, 2022). Taken together, these considerations suggest that healthcare professionals’ representations of aging emerge from the interplay of personal, professional, organizational, and broader socio-cultural experiences. Despite the relevance of these dynamics, relatively little is known about how old age is represented by healthcare professionals, particularly in Southern European contexts.

### Theoretical Framework

The present study is grounded in a psychosocial and cultural–psychological perspective that conceptualizes old age as a socially and culturally embedded phenomenon, rather than as a condition defined solely by individual characteristics or chronological age. Within this framework, representations of old age are understood, following Moscovici (2003), as socially constructed systems of meaning through which individuals make sense of their experiences and of the broader social world. Such representations develop through interpersonal communication and emerge from the interplay between personal, social, and professional experiences, shaping how old age is perceived, interpreted, and related to. Therefore, for healthcare professionals working with older adults, the meanings attributed to old age may therefore emerge at the intersection of professional encounters with older people and personal, family, and broader sociocultural experiences. Their accounts may bring together professional, personal, and socially shared ways of making sense of old age, without these different sources necessarily being distinguishable from one another. Accordingly, the aim of the present study was not to isolate a specific professional representation of old age, but to explore the configurations of meaning emerging within the discourse of a specific professional group.

This conceptualization also informed the choice of the interview format. The use of a single open-ended question allowed participants to organize their accounts around the experiences and associations that were most salient to them, rather than around predefined dimensions of aging. Such an approach was consistent with the view of representations as dynamic systems of meaning emerging through communication and shaped by multiple domains of experience.

Given this socio-constructivist conceptualization of old age, its exploration requires a method capable of capturing not only explicit and rational definitions, but also the latent, affectively connoted, and socially shared meanings through which later life is interpreted. For this reason, the present study adopted Emotional Text Mining (ETM) as its methodological framework. ETM is an unsupervised text-mining methodology grounded in a socio-constructivist perspective and a psychodynamic model of meaning-making (Greco & Polli, 2021). It was considered particularly suitable for the aims of this study because it enables the analysis of associative patterns emerging from participants’ language without imposing predefined interpretative categories.

Unlike approaches that begin by assigning textual material to an established coding framework, ETM first reconstructs the statistical organization of lexical co-occurrences across the corpus and subsequently proceeds to the interpretative labeling of the structures identified. Within this framework, recurrent patterns of word selection and co-occurrence are understood not merely as linguistic regularities, but as indicators of the culturally shared representations through which a social group organizes and gives meaning to a specific object. The term “emotional” refers to the affectively connoted processes through which experience is symbolized and categories of meaning are organized. Lexical co-occurrences are therefore considered empirical traces, rather than direct measures, of the shared symbolic organization of the object under investigation. Although the narratives could have been examined through different qualitative approaches, the choice of ETM was guided by the specific level at which the research question was formulated. More specifically, the purpose of the present study was to map the shared semantic field emerging across the corpus by identifying recurrent discursive configurations and the principal symbolic oppositions through which old age became collectively meaningful within this specific professional group. The study therefore aimed to explore, through the ETM method, how healthcare professionals make sense of old age through their personal, social, and cultural experiences, including those developed through their professional work. Accordingly, the following research questions were formulated:

Q1: What representations of old age emerge from healthcare professionals’ narratives?
Q2: Are the distributions of these representations across the identified clusters associated with participants’ age, gender, and location (that is city of residence and professional practice)?

Understanding these dynamics has significant implications: (1) at the individual level, it can guide the design of training programs that promote more reflective approaches to geriatric care; (2) at the social level, it contributes to a broader cultural debate on how we collectively imagine and value old age.

## 2. Materials and Methods

### 2.1. Transparency and Openness

This study is part of a larger research project “Development and testing of psycho-promotion indicators for a successful aging of older adults” planned and conducted by the Department of Human and Social Sciences at the University of Bergamo and the Department of Dynamic and Clinical Psychology and Health Sciences at Sapienza University of Rome. The study was approved by the Ethics Committee of the University of Bergamo (protocol code 2024_05_03, date of approval 17 May 2024), in accordance with the principles of the Declaration of Helsinki and the Convention on Human Rights and Biomedicine (Oviedo Convention) and all participants provided informed consent.

Anonymized data supporting the findings of this study are available from the authors upon reasonable request, due to privacy and confidentiality constraints.

The study design, hypotheses, and analytic plan were not preregistered. The reporting of the study was reviewed against the Consolidated Criteria for Reporting Qualitative Research (COREQ) 32-item checklist. The complete COREQ checklist is provided in the Supplementary Material (See Table S1).

### 2.2. Participants and Procedure

This study employed an exploratory qualitative design based on individual, in-person interviews. Verbatim interview transcripts were analyzed using a quali-quantitative approach based on ETM methodology (see Section 2.3).

Men and women living and practicing in Rome and Bergamo were eligible to participate if they met the following inclusion criteria:

- Currently working as healthcare or allied health professionals in geriatric care;
- Aged between 30 and 60 years.

This age range was selected to include professionals at different stages of adult life, allowing for variation in personal, social, and professional experiences and enabling potential age-related and working experience-related differences in representations of old age to be explored within a relatively comparable working-age group.

Participants were recruited through purposive and snowball sampling to ensure an equivalent representation across both geographical contexts. In both cities, the same recruitment approach was applied: local residential care facilities were contacted, with three facilities involved in Bergamo and five in Rome, aiming to recruit matching professional profiles in each setting (i.e., psychologists, neurologists, neuropsychologists, and healthcare assistants). Initial participants were identified through professional networks and the selected healthcare services involved in geriatric care and were subsequently asked to indicate other potentially eligible professionals. Overall, approximately half of the participants in each city were recruited through snowball sampling, with a slightly higher proportion recruited through this strategy than through purposive sampling in both contexts.

Potential participants were contacted by telephone by a psychologist responsible for implementing the study protocol. Those who agreed to participate were invited to attend an appointment during which they completed the brief sociodemographic questionnaire and took part in the interview. Before completing the questionnaire and participating in the interview, all participants provided written informed consent. Each participant took part in a single face-to-face interview conducted in a private setting, with no other people present. The interviews were conducted by two female psychologists, both holding a master’s degree in psychology and having previous experience in conducting qualitative interviews; both were co-authors of the study (A.G. and M.G.). Neither interviewer had a prior relationship with any of the participants.

The single open-ended question used in the present study to elicit participants’ narratives about their representations of old age was: “As researchers, we are trying to understand the way people cope with old age: the resources they can draw on, the difficulties they face, the way they relate to this transition. We are very interested in hearing about your opinion and experience”.

After asking the initial question, the interviewer used additional prompts—such as brief echo responses—for the sole purpose of facilitating the interviewee’s narrative exploration and only when the participant’s silence suggested a possible premature conclusion to the response, thus avoiding interrupting or steering the flow of the conversation. The prompt did not explicitly require participants to focus on their professional practice. Participants were free to draw on personal, family, social, and work-related experiences, all of which were included in the corpus. The average duration of each interview was approximately 30 min, with durations ranging from 19 to 38 min. No field notes were taken. Neither the transcripts nor the findings were returned to participants for comment or validation. At the end of the meeting, essential socio-demographic data (age, gender, city of residence and professional practice, and professional role) were collected.

Data collection occurred between October 2024 and February 2025.

The sample size was determined a priori at 50 participants to obtain two equally sized subsamples—25 participants from Rome and 25 from Bergamo—and thereby support the planned comparison of cluster distributions between the two locations. This was a design-based decision; no formal statistical power calculation was performed, and data saturation was not used as a recruitment stopping criterion and all collected data were included in the analyses; no data was excluded. Given the corpus-based nature of ETM, the adequacy of the textual material for statistical analysis was subsequently assessed through lexical indicators, including the type–token ratio and the percentage of hapaxes.

During the recruitment phase, three professionals declined to participate; therefore, 53 professionals were contacted to obtain the planned sample of 50 participants (see Table 1 for full socio-demographic characteristics).

The gender distribution in the present study is consistent with that of the target population, which is characterized by a greater proportion of women (Istituto Nazionale di Statistica, 2026; Ministero della Salute, 2021).

### 2.3. Data Analysis

All interviews were audio-recorded and transcribed verbatim and collected in a textual corpus. The analyses were performed on the original Italian-language transcript using T-LAB software, version 10.2.6. The corpus was analyzed using the Emotional Text Mining (ETM) procedure (Greco, 2016; Greco & Polli, 2021) a bottom-up, unsupervised methodology for natural language processing based on a socio-constructivist and psychodynamic approach and widely applied in psychology and social sciences (Di Trani et al., 2021).

Corpus preprocessing was performed automatically in T-LAB following the procedure described by Cordella et al. (2014). Using its Italian-language resources, the software performed text normalization, identification of multiword expressions, vocabulary construction, lemmatization, and segmentation of the corpus into context units (CUs) based on punctuation, segment length, and statistical criteria. T-LAB also automatically excluded stop words and non-content grammatical categories, including articles, prepositions, conjunctions, adverbs, pronouns, and adjectives, while retaining nouns and verbs as content-bearing lexical units. No manual modification of multiword expressions or lemma assignments was performed. Lexical units were subsequently selected according to their corpus frequency. The minimum frequency threshold was set at eight occurrences, corresponding to the total number of categories across the illustrative variables included in the analyses: four age categories, two location categories, and two gender categories (Greco, 2016). At the upper end of the frequency distribution, lexical units belonging to the high-frequency rank were excluded because they were considered ubiquitous and insufficiently discriminating within the corpus. Following Bolasco (2010), the boundary between the high- and medium-frequency ranks was identified from the descending frequency distribution as the first frequency value shared by more than one lexical unit; frequency values above this boundary, which occurred only once in the distribution, constituted the high-frequency rank. The final lexical set comprised 365 keywords.

The preprocessing procedure generated 1419 context units. Of these, 1409 (99.3%) contained at least two co-occurring selected lexical units and were retained for analysis; the remaining 10 CUs were excluded. Each interview transcript corresponded to a single participant and was tagged with that participant’s age group, gender, and location before segmentation. Consequently, each context unit retained the illustrative-variable labels associated with its source transcript. Two indicators of lexical richness were calculated—the type–token ratio (TTR) and the percentage of hapaxes—to assess the suitability of the corpus for statistical analysis.

A term matrix for CUs was created from the 1409 retained context units and subjected to a bisecting k-means cluster analysis based on cosine similarity (Savaresi & Boley, 2004). Cluster solutions were limited to 20 partitions, excluding CUs with fewer than two co-occurring terms. The optimal number of clusters was identified by examining intraclass correlation coefficient (ρ) calculated as the ratio between-cluster variance and total variance, whereas the Gap represents the increase in the Index relative to the immediately preceding partition. Calinski–Harabasz, Davies–Bouldin, and ICC (rho) values were additionally obtained for the retained partition (Misuraca et al., 2019).

Each cluster represents a discursive nucleus, i.e., a thematically and symbolically coherent area of the corpus.

To explore the latent symbolic structure that organizes the clusters, a correspondence analysis (CA) was performed on the cluster-by-term matrix (Lebart et al., 1997). While cluster analysis identifies thematic representations, CA reveals the deeper oppositional dimensions (latent and representable as identifiable meanings through the opposition of their extremes)—called symbolic factors—through which the narrative space is polarized (Greco, 2016; Greco & Polli, 2021). These dimensions do not express value judgments but map contrasting positions within the symbolic field.

The interpretation followed the logic of ETM, which reconstructs symbolic–emotional structures from surface linguistic data, mirroring the reverse of the cognitive process through which meaning is produced (Di Trani et al., 2021). The interpretative procedure involved three independent judges with backgrounds in psychology and specific expertise in ETM: an associate professor, a researcher, and a psychologist with specialist training in the methodology. The judges initially worked independently. Each judge examined the lexical units associated with the positive and negative poles of the factors, their absolute contributions and coordinates, the characteristic lexical units of each cluster, and the position of the clusters within the factorial space. On this basis, each judge independently proposed preliminary labels and interpretations for the factors and clusters. During this independent stage, the judges were blinded to participants’ age, gender, and location, and the illustrative variables were not used to formulate the preliminary labels. The independent interpretations were subsequently compared in a joint discussion. When differences emerged, the judges returned to the lexical units, their statistical contributions and coordinates, and the position of the clusters within the factorial space. Interview excerpts were consulted only during this subsequent consensus phase to contextualize, compare, and progressively refine the proposed interpretations. This process was iterative and continued until the judges reached a shared interpretation and agreed on the final labels.

No formal inter-rater agreement coefficient was calculated. The judges generated open-ended interpretative labels rather than assigning units to predefined categories; therefore, the robustness of the interpretative process was pursued through independent analysis followed by iterative, evidence-based discussion and consensus.

Finally, chi-square tests were performed at the context-unit level to explore whether the distribution of context units across the identified clusters was associated with participants’ age, gender, and location. Each context unit inherited the illustrative-variable characteristics of the participant from whose interview it originated. Because each participant contributed multiple context units, these analyses were conducted at the context-unit level and should not be interpreted as direct participant-level comparisons. The nesting of context units within participants was not explicitly modeled; therefore, the findings from these analyses were considered exploratory and interpreted with caution. For this analysis, age was grouped into four approximately equal-sized categories based on the distribution of participants’ ages: 30–45, 46–49, 50–57, and 58–60 years. This categorization was adopted to obtain groups with adequate and broadly comparable representation; the cut-off points were not intended to reflect theoretically predefined stages of adulthood (Capogna & Greco, 2024). For all participants, city of residence and city of professional practice coincided; these were therefore operationalized as a single location variable with two categories: Rome and Bergamo. Professional roles were collected solely to describe the composition of the sample and were not included as an illustrative variable in the statistical analyses, because the small and uneven frequencies across occupational categories did not permit reliable comparisons between professional groups. To account for multiple testing, a Bonferroni-adjusted significance threshold of α = 0.0167 was applied to the three tests. Pearson residuals were used descriptively to identify the context-unit cells contributing most strongly to a significant overall association.

In the presentation of the results, clusters refer to coherent discursive configurations identified through patterns of lexical co-occurrence, rather than to profiles assigned to individual participants, whereas factors refer to the underlying oppositional dimensions that organize the semantic space. Accordingly, each cluster is interpreted by considering both its characteristic vocabulary and its position within the factorial space. Consistent with the socio-constructivist framework of ETM, the analyses involving illustrative variables were intended to explore how context units associated with different participant groups were distributed across the symbolic field, rather than to classify participants or estimate the prevalence of individual-level representations.

## 3. Results

The transcription of the interviews generated a corpus of 72,293 tokens, comprising 6899 types. The lexical indices indicated the feasibility of performing Emotional Text Mining: the TTR was 0.095, well below the 0.20 threshold typically used for evaluating lexical richness in large corpora, while hapax legomena accounted for 50.16% of the lexicon (3560 hapax).

The segmentation process produced a set of comparable text chunks. Overall, 1419 context units (CUs) were generated, of which 1409 (99.3%) were retained for analysis.

Correspondence analysis identified six latent factors organizing the semantic space in which clusters are located. The explained inertia for each factor is reported in Table 2. However, following the standard methodological criteria of Emotional Text Mining (Greco & Polli, 2021) and the empirical approach consolidated in the literature (Di Trani et al., 2021) the discussion will focus exclusively on the first three factors by balancing the amount of inertia represented against the complexity of the factorial solution. This choice is justified by the principle of statistical parsimony: these dimensions account for the largest share of total inertia (equal to 60.24%), identifying the macro-affective polarizations and the main symbolic matrices of the corpus. Together, they therefore provide a parsimonious low-dimensional representation that preserves most of the association structure without introducing the interpretative complexity of a six-dimensional solution. Retaining three factors also makes it possible to consider their relationships jointly within a single three-dimensional semantic space, facilitating the visualization and interpretation of the relative positions of the clusters. Factors 4-6 are reported in Table 2 for transparency but were not interpreted in depth, as their additional contribution was considered insufficient to offset the increased complexity of a higher-dimensional representation.

### 3.1. The Factors

Factor 1 accounted for 23.52% of total inertia. Its negative pole was primarily characterized by sons (percentage contribution = 3.16%), work (2.33%), get out (1.64%), grown-up (1.38%), return (1.00%), sport (0.96%), grandchild (0.80%), COVID-19 (0.74%), university (0.74%), and partner (0.69%), whereas its positive pole was characterized by think (3.9%), difficulties (2.95%), people (0.95%), future (0.86%) discourse (0.53%), build (0.43%), face (0.42%), practice (0.40%), accept (0.36%), and lose (0.34%).

Factor 2 accounted for 20.11% of total inertia. Its negative pole was primarily characterized by home (percentage contribution = 1.62%), beautiful (1.18%), succeed (1.16%), talk (1.13%), pleasure (0.62%), child (0.53%), care for (0.49%), possibility (0.47%), wash (0.45%), and group (0.38%), whereas its positive pole was characterized by ageing (6.50%), resources (3.07%), retirement (2.28%), old age (1.35%), cultural (1.01%), concern (0.95%), pathology (0.79%), limits (0.79%), risk (0.66%), and expectation (0.64%).

Factor 3 accounted for 16.61% of total inertia. Its negative pole was primarily characterized by years (percentage contribution = 24.55%), older adult (2.72%), beginning (1.85%), mother (1.46%), young (0.93%), residential care facility (RSA; 0.80%), adult (0.68%), bar (0.66%), change (0.66%), enjoy (0.55%), and father (0.55%), whereas its positive pole was characterized by find (2.77%), activities (1.20%), study (0.95%), relationships (0.95%), call (0.81%), pathway (0.78%), organize (0.75%), pay (0.68%), context (0.65%), and ability (0.62%).

### 3.2. The Clusters

**Figure 1:**
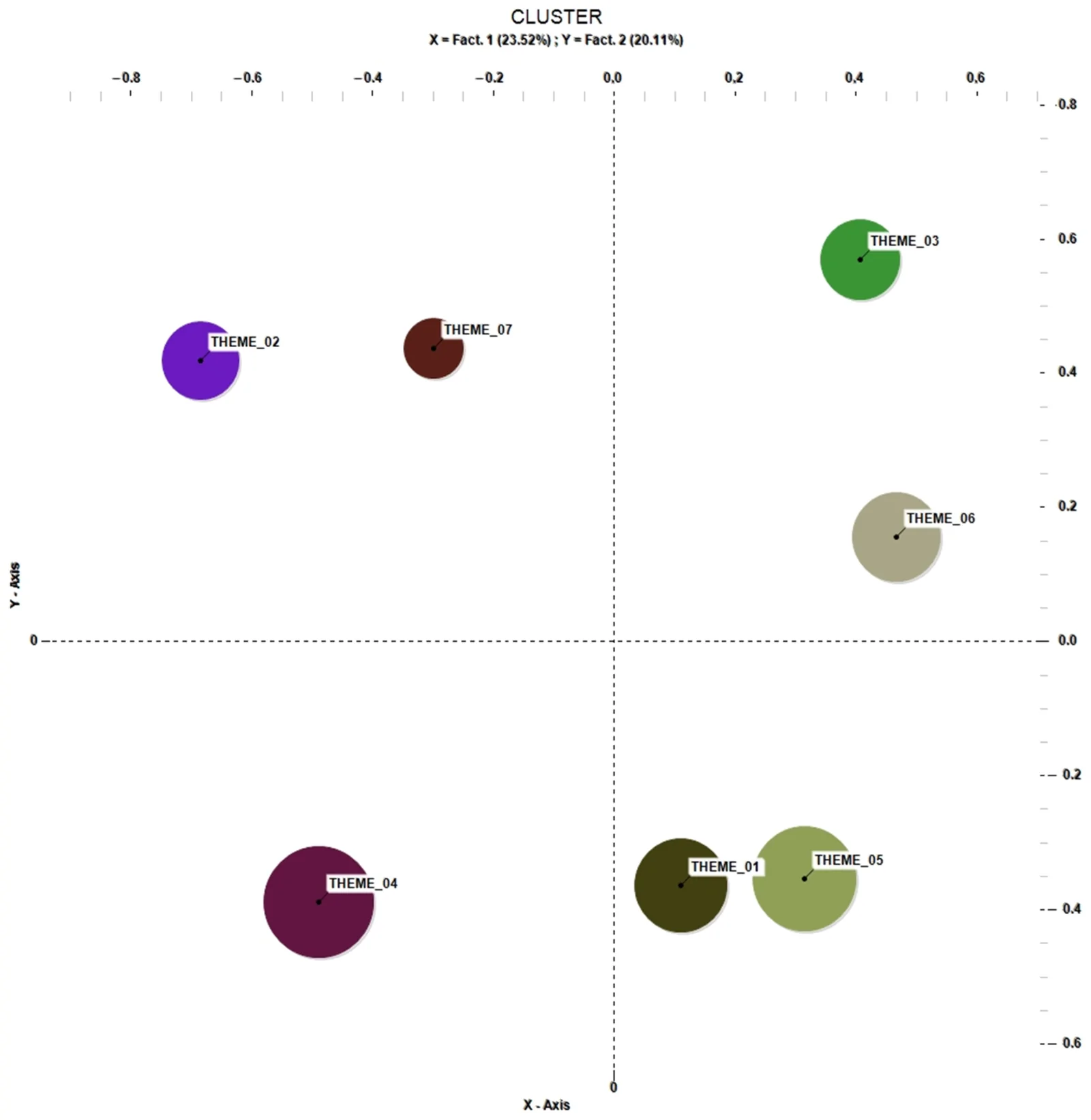
Projection of the seven clusters onto the factorial plane defined by Factor 1 (x-axis; 23.52% of total inertia) and Factor 2 (y-axis; 20.11% of total inertia). Note. Each colored circle represents one of the seven clusters (CL_01–CL_07). Colors are used consistently across Figure 1 and Figure 2 to distinguish the clusters and have no additional statistical meaning. Circle size reflects the relative number of context units assigned to each cluster, while cluster position indicates its relationship to the positive and negative poles of the two factors.

**Figure 2:**
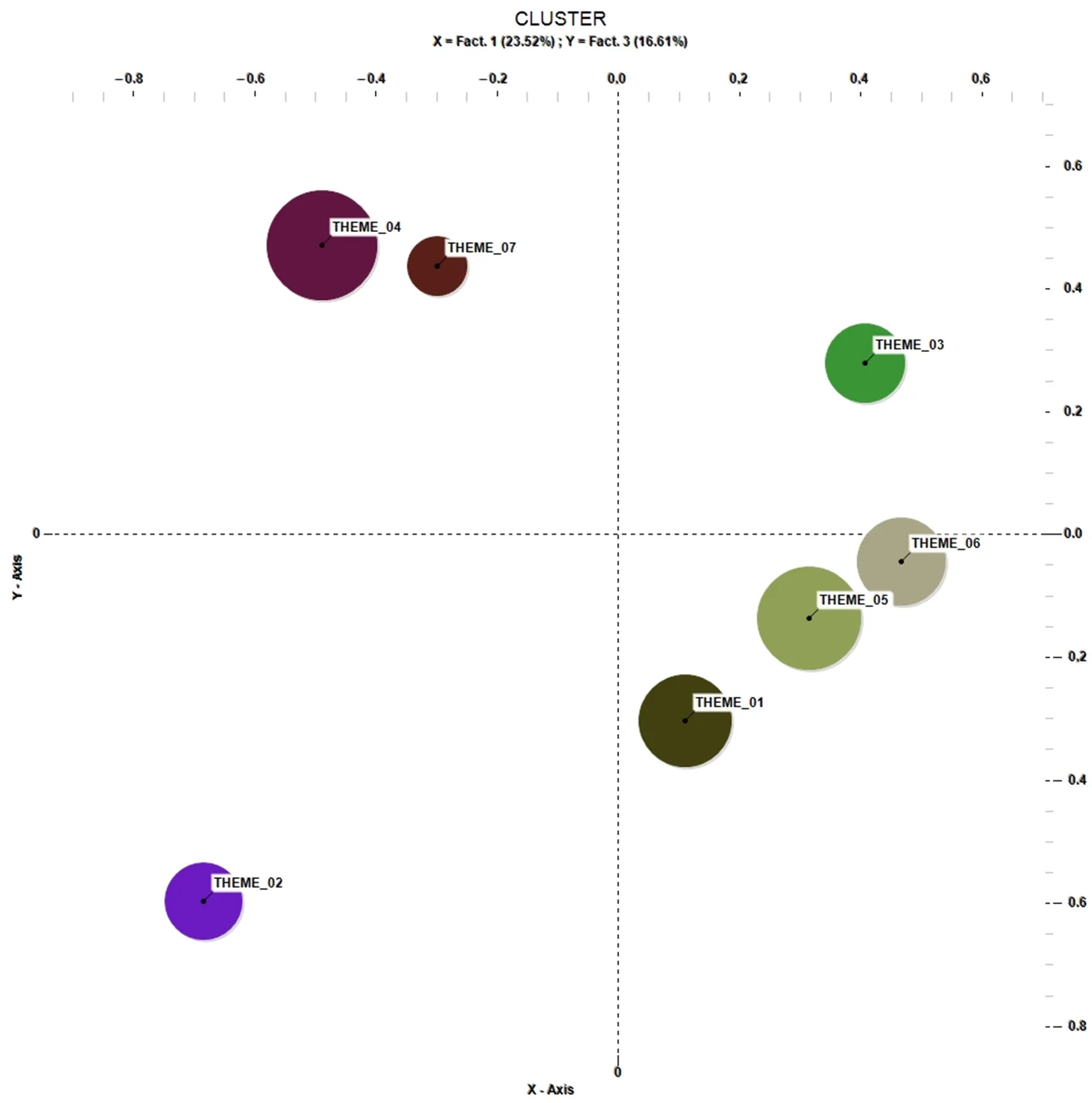
Projection of the seven clusters onto the factorial plane defined by Factor 1 (x-axis; 23.52% of total inertia) and Factor 3 (y-axis; 16.61% of total inertia). Note. Each colored circle represents one of the seven clusters (CL_01–CL_07). Colors are used consistently across Figure 1 and Figure 2 to distinguish the clusters and have no additional statistical meaning. Circle size reflects the relative number of context units assigned to each cluster, while cluster position indicates its relationship to the positive and negative poles of the two factors.

The placement of the clusters in the factorial space is illustrated in Table 3 and Figure 1 and Figure 2.

Before presenting the individual clusters, the available cluster partitions were compared using the Index (intraclass correlation coefficient) and Gap values provided by T-LAB. In this output, the Index corresponds to ICC (rho), whereas the Gap represents the incremental increase in the Index relative to the preceding partition. The two-, three-, four-, five-, six-, seven-, eight- and nine-cluster solutions yielded Index values of 0.0081, 0.0201, 0.0350, 0.0535, 0.0725, 0.0955, 0.1161, and 0.1375, respectively. The corresponding Gap values were 0.0000, 0.0120, 0.0149, 0.0185, 0.0190, 0.0230, 0.0206, and 0.0213. Although the Index increased as additional clusters were introduced, the incremental improvement reached its maximum for the seven-cluster solution: the Gap increased from 0.0190 at six clusters to 0.0230 at seven clusters and then decreased to 0.0206 at eight clusters. According to T-LAB’s selection criterion, the seven-cluster partition, the solution immediately preceding the decrease in the Gap, was therefore identified as the best-fitting solution. For the retained seven-cluster partition, the additional quality indices were 24.684 for the Calinski–Harabasz index, 1.352 for the Davies–Bouldin index, and 0.096 for ICC (rho). The seven-cluster solution was selected by considering these quantitative values together with the semantic coherence and interpretability of the resulting clusters. The selected partition comprised seven thematic clusters, capturing the main symbolic and emotional areas associated with representations of ageing.

Cluster 1 comprised 217 context units, corresponding to 15.4% of the analyzed corpus. The ten lexical units most strongly associated with the cluster were see (χ^2^ = 650.48), maintain (χ^2^ = 34.12), different (χ^2^ = 27.22), approach (χ^2^ = 21.52), time (χ^2^ = 21.42), older adult (χ^2^ = 20.06), male (χ^2^ = 19.88), succeed (χ^2^ = 12.95), staff (χ^2^ = 12.13), and wash (χ^2^ = 12.04); all associations were statistically significant at *p* ≤ 0.001.

*Changes happen—even the older people themselves arrive here in one condition, then you see the decline many times, because maybe before they greeted you, you were able to talk to them, then maybe for various reasons—health-related things and so on—you really see… there is really like a fall, until you arrive in the morning and they tell you that they’re no longer here, that maybe… but you know it. (male, Bergamo, SCORE 406.429)*

Cluster 2 comprised 177 context units, corresponding to 12.6% of the analyzed corpus. The ten lexical units most strongly associated with the cluster were years (χ^2^ = 1021.25), start (χ^2^ = 88.12), mother (χ^2^ = 48.00), age (χ^2^ = 42.68), peer (χ^2^ = 33.98), retirement (χ^2^ = 29.11), sport (χ^2^ = 21.84), professional (χ^2^ = 20.33), COVID-19 (χ^2^ = 19.93), and children (χ^2^ = 19.72); all associations were statistically significant at *p* < 0.001.

*Whereas, for example, my peers work—that is, they already start thinking about retirement in 15 years, in 18 years, or my peers… I was born in ’76, I’m 48 years old, so let’s say that my friends who started working very young—because, for example, they worked in the armed forces and so they passed the exams at 18–19 years old, when you used to do military service, and they… (male, Bergamo, SCORE 1501.618)*

Cluster 3 comprised 180 context units, corresponding to 12.8% of the analyzed corpus. The ten lexical units most strongly associated with the cluster were the verb age (χ^2^ = 289.50), resources (χ^2^ = 114.02), cultural (χ^2^ = 51.63), pathology (χ^2^ = 35.94), respond (χ^2^ = 32.99), draw on (χ^2^ = 32.64), propose (χ^2^ = 32.64), question (χ^2^ = 26.27), complex (χ^2^ = 24.33), and discourse (χ^2^ = 23.79); all associations were statistically significant at *p* < 0.001.

*The resources that one can draw upon when facing aging—there must be a clear distinction between aging well, aging in good health, or aging with illness. When aging involves illness, the resources available today exist in terms of assistance (male, Bergamo, SCORE 496.535).*

Cluster 4 comprised 259 context units, corresponding to 18.4% of the analyzed corpus. The ten lexical units most strongly associated with the cluster were find (χ^2^ = 138.21), work (χ^2^ = 99.39), go out (χ^2^ = 80.44), home (χ^2^ = 55.15), return (χ^2^ = 44.16), beautiful (χ^2^ = 43.91), children (χ^2^ = 42.99), small (χ^2^ = 42.63), study (χ^2^ = 38.86), and relationships (χ^2^ = 37.65); all associations were statistically significant at *p* < 0.001.

*Other things, uh… No, above all, I think this is another important thing… trying—which is fundamental—to maintain a balance between work activity, family life, social life, that is, not letting… not being completely absorbed by work, even if it’s work you like. It’s necessary to find your own spaces… (female, Rome, SCORE 145.888)*

Cluster 5 comprised 244 context units, corresponding to 17.3% of the analyzed corpus. The ten lexical units most strongly associated with the cluster were need (χ^2^ = 233.78), time (χ^2^ = 94.72), older adult (χ^2^ = 79.16), understand (χ^2^ = 59.11), family (χ^2^ = 53.41), realize (χ^2^ = 43.77), talk (χ^2^ = 31.56), fear (χ^2^ = 30.82), listen (χ^2^ = 30.78), and language (χ^2^ = 26.21); all associations were statistically significant at *p* < 0.001.

*Surely an older person must in some way be socially recognized, and in my opinion this is not the case. This opens up a series of issues—I often think of the phrase “do whatever you want and time will pass.” We need to rethink the role of the older adult; we need to open ourselves to complexity. (male, Rome, SCORE 222.374)*

Cluster 6 comprised 202 context units, corresponding to 14.3% of the analyzed corpus. The ten lexical units most strongly associated with the cluster were think (χ^2^ = 199.55), services (χ^2^ = 194.46), difficulties (χ^2^ = 74.76), hospital (χ^2^ = 55.60), demand (χ^2^ = 37.08), meet (χ^2^ = 29.87), app (χ^2^ = 29.17), community (χ^2^ = 28.51), network (χ^2^ = 20.89), and management (χ^2^ = 20.25); all associations were statistically significant at *p* < 0.001.

*Well, then, if I think about the hospital context where I work, well, geriatric services are missing—in the hospital they are practically disappearing. If I think instead about the issue of community setting, I think of the houses, that is, the Community Hospitals. (Male, Rome, SCORE 416.179)*

Cluster 7 comprised 130 context units, corresponding to 9.2% of the analyzed corpus. The ten lexical units most strongly associated with the cluster were problem (χ^2^ = 117.72), cognitive (χ^2^ = 80.49), retirement (χ^2^ = 79.37), theme (χ^2^ = 69.32), system (χ^2^ = 55.04), social (χ^2^ = 39.74), related (χ^2^ = 36.69), age (χ^2^ = 33.27), critical issue (χ^2^ = 31.68), and energy (χ^2^ = 29.80); all associations were statistically significant at *p* < 0.001.

*The limitations are mostly physical rather than cognitive; there may be neurological problems involving the lower limbs, tremors that don’t allow them to sit at the table calmly with relatives if they go out, or that limit their activities with others, or that make them feel ashamed. Cognitive limitation does not allow awareness of one’s own condition. (female, Rome, SCORE 107.227)*

### 3.3. Different Representation of Old Age Across Clusters According to the Considered Socio-Demographic Variables

Chi-square tests were conducted at the context-unit level to examine whether cluster distribution differed according to participants’ age, gender, and location. To account for multiple testing, a Bonferroni-adjusted significance threshold of α = 0.0167 was applied to the three tests. No statistically significant differences were found regarding age or gender. Location was significantly associated with cluster distribution (χ^2^ = 49.84; df = 6; *p* < 0.001). The effect size indicated a small association (Cramér’s V = 0.19). The contingency table for this significant association is reported in Table 4. All expected frequencies were well above 5, with the lowest expected frequency being 42.35. Examination of the Pearson residuals showed that context units originating from the Rome interviews occurred more frequently than expected in Clusters 5 (Awareness) and 6 (Rethinking Services) and less frequently than expected Clusters 2 (The Passing Years) and Cluster 4 (Denial). The opposite descriptive pattern was observed for context units originating from the Bergamo interviews.

## 4. Discussion

The aim of the present study was to explore representations of old age among healthcare professionals working with older adults and to examine whether the distribution of these representations was associated with participants’ age, gender, and location.

Because the corpus included personal, family, social, and professional experiences, these configurations should not be attributed exclusively to professional experience but understood as reflecting an intersection of meanings drawn from these different domains. With the idea of creating a taxonomy of the cultural and affective constructions of old age among healthcare workers who work in the elderly field, we mapped the semantic field, highlighting its symbolic polarities, areas of greater density, and less frequently occupied regions of the discursive space.

Although T-LAB first identifies the clusters and subsequently performs correspondence analysis, for explanatory and interpretative purposes the findings are discussed here in the reverse order. We first consider the field of meanings delineated by the factors and then examine how the clusters are positioned within this semantic space. Accordingly, the factors are initially treated as bipolar semantic dimensions defined by the lexical units characterizing their negative and positive poles, whereas the clusters are subsequently interpreted in relation to their placement within the factorial space.

At its negative pole, Factor 1 was characterized by lexical units referring to family relationships (sons, grandchild, partner), work and education (work, university), and activities involving movement or continuity in everyday life (get out, return, sport). Taken together, these words may be interpreted as anchoring ageing in the present. By contrast, the positive pole included the explicit term future, together with difficulties and a prevalence of verbs—think, build, face, accept, and lose—that evoke anticipation and the effort to imagine and confront what lies ahead. Based on this opposition, Factor 1 was labelled “Time”, articulated between a “Present” grounded in ongoing relational and everyday experience and a “Future” represented as something to be constructed and faced, but also marked by possible difficulty and loss.

At its negative pole, Factor 2 was characterized by words referring to a familiar, domestic, and relational context, including home, beautiful, talk, pleasure, child, care for. These lexical units may be interpreted as evoking a symbolically protected space. By contrast, the positive pole included old age, cultural, concern, pathology, limits, risk, and expectation, which foreground vulnerability, uncertainty, and the potentially threatening implications associated with ageing. Since this opposition, Factor 2 was labelled “Places”, articulated between a “Protected Shelter” and “Exposure to Risk”.

At its negative pole, Factor 3 was strongly characterized by years, together with lexical units marking different stages in the life course (older adult, beginning, young, adult, change), family figures (mother, father). These words may be interpreted as anchoring in situations in which care is provided, received, or incorporated into everyday life. By contrast, the positive pole included find, activities, study, relationships, call, pathway, organize, pay, context. This lexical pattern foregrounds searching, coordinating, and mobilizing activities, relationships, abilities, and practical resources, suggesting a more prospective orientation toward constructing possible pathways and organizing responses. On the basis of this opposition, Factor 3 was labelled “Care”, articulated between “Being involved in care” and “Building one’s own life project”. 

More broadly, these dimensions suggest that old age is represented through three interconnected questions: how it is positioned between present experience and future anticipation, where it is imagined between protection and exposure, and how care is conceived between assistance and planning. Within this semantic space, the seven clusters represent the main discursive configurations through which these tensions are articulated, outlining the different ways in which later life is conceptualized in participants’ discourse. This relatively high number of word groupings seems to suggest that the topic is particularly complex and multifaceted. The complexity and granular structure of textual data, when healthcare workers are questioned on this subject, also appeared evident in other similar studies concerning the process of social representation of old age (e.g., Coelho et al., 2025). Overall, the findings indicate that the corpus does not convey a single coherent image of later life. Instead, from an interpretative perspective, participants’ discourse may be read as moving between the acceptance of aging as part of the life course, defensive distancing from its emotional implications, relational engagement with older adults, and different forms of individual or institutional action.

Considering the first and second factors together (that defining the main factorial plane), clusters 3 “Challenging old age” and 6 “Rethinking services” are in the quadrant defined by the poles “Future” and “Exposure to Risk.” Both identify a semantic area centered on intervention and action, but they differ in terms of the question that guides this intervention. In cluster 6, “Rethinking services,” the call for action appears to be directed primarily at the social and institutional system, as suggested by words such as services, hospital, demand, community, network, and management. From an interpretative perspective, improving services may be understood as serving a dual function: strengthening the effectiveness and continuity of care while making its practical and emotional burden more collectively sustainable. When read through a rights-based lens, it may also position older adults as citizens and subjects of law, rather than merely as recipients of health and social assistance, thereby linking the representation of old age to a process of legal and social recognition already observed in relation to other vulnerable groups (Paniccia, 2023).

In cluster 3, on the other hand, “Challenging old age,” words such as resources, pathology, respond, draw on, propose, and complex suggest an orientation toward mobilizing resources and constructing responses to the complexity of ageing. The cluster is also located toward the positive pole “Building one’s own life project” of the third factor. Consistent with this lexical pattern and its factorial position, old age may be interpreted as a challenge to be actively confronted rather than passively endured. This interpretation provides a possible counterpoint to stereotypes that reduce later life to decline and dependence. At a theoretical level, it resonates with active and successful aging frameworks, which emphasize autonomy, resilience, and the individual management of age-related risks (Rowe & Kahn, 1997). As critical gerontology has observed, however, such frameworks may also risk obscuring the social and structural conditions that shape aging, turning vulnerability into a primarily individual responsibility (Katz, 2001).

Clusters 1 (“Witnessing the Aging Process”) and 5 (“Awareness”) are also located on the Future side of the first factor, but within the Protected Shelter pole of the second. Unlike the action-oriented configurations described above, these clusters may be interpreted as expressing an exploratory and relational orientation: aging may be approached not primarily as a risk to be controlled, but as an experience to be understood through sustained contact with older adults. In Cluster 1 words such as see, maintain, different, approach, time, and older adult support the interpretation of a form of knowledge grounded in witnessing changes in older adults over time. Through the continuous observation of gains, losses, adaptation, and decline, participants seem invited to recognize the older person as a subject. When viewed through the theoretical lens of mentalization this process may be understood in relation to the capacity to recognize and differentiate one’s own mental states from those of others (Fonagy et al., 2018). Mentalization is used here exclusively as a theoretical lens for interpreting the lexical configuration; it was not directly assessed and should not be understood as a construct empirically demonstrated by the present study. Within this theoretical reading, seeing may be understood not only as physical observation but also as psychic recognition. Through a Winnicottian lens, the effort to contain, think about, and accept the subjectivity of others may also be interpreted in terms of mirroring and attunement (Winnicott, 2018). Although ageism was not directly assessed in the present study, this interpretation is consistent with evidence showing that emotional closeness and physical proximity, together with knowledge of the aging process (in particular, the extreme variability of health conditions and psychological and socio-cultural resources available to older adults), are powerful means of counteracting ageism (Burnes et al., 2019; Sarabia-Cobo & Pfeiffer, 2015).

Alongside this mode of knowledge based on testimony, Cluster 5, “Awareness,” whose characteristic words include need, time, older adult, understand, family, realize, talk, fear, listen, and language, may be interpreted as introducing a representation of old age mediated by a comparison with what being old implies from a relational point of view, a change that appears inevitable. The fact that this cluster is associated with the pole relating to “Being involved in care” may be read as reflecting an awareness, potentially drawing on their personal, professional and social experience, that ageing implies new needs, primarily related to language and listening, relational vehicles that may become more salient with the slowing down of action. Taken together, clusters 1 and 5 may be interpreted as describing a shift from knowing about old age to knowing older people through the care relationship. In these interpretative reading, the distance between observer and observed may be partially reduced, at least in part, because in both healthcare clusters they seem to be involved in an interpersonal relationship with the older adult, who are considered privileged interlocutors.

On the Present side of the Time factor, Clusters 2, 4, and 7 may be interpreted as revealing different ways of responding to the immediacy of aging and to the limits it makes visible. Cluster 4 (“Denial”) is located within the Protected Shelter pole, whereas Clusters 2 (“The Passing Years”) and 7 (“The Problem of Old Age”) are associated with Exposure to Risk. This contrast suggests that an orientation toward the present does not have a single meaning: it may support defensive distancing from aging, its integration into the continuity of the life course, or its critical problematization. In Cluster 4, “Denial,” words such as find, work, go out, home, return, beautiful, children, study, relationships, and pleasure evoke continuity with everyday activities, relationships, and sources of enjoyment. From an interpretative perspective, this lexical pattern may be read as indicating lack of space, in the present, to feel the emotions linked to one’s own aging. The label “Denial” therefore represents an interpretation of the lexical configuration rather than a psychological process directly assessed by the study. The cluster is also located in proximity to the positive pole of Factor 3, “Building one’s own life project” which is consistent with its emphasis on continued investment in everyday life.

Continuing with the narrative, participants’ discourse shifts their focus to the inevitability of the passing of time and the role assigned to the older adult by the socio-cultural context”. Cluster 2 seems to describe precisely this dimension, as suggested by words such as years, start, mother, age, peer, retirement, professional, and children, probably influenced by its spatial proximity to the negative pole of Factor 3, “Being involved in care”. Within this configuration, old age may be interpreted not as an exceptional state or a condition of lack of planning, often associated with an idea of economic and productive “uselessness”, but rather as a natural phase of the life cycle. When read through a life-course lens, this perspective is consistent with Erikson’s reflection on the last stage of psychosocial development, in which the individual is called upon to integrate their life history by confronting finitude and the meaning of their existence (Erikson & Erikson, 1998), and with Boggi Cavallo and Cesaro (2023) idea of old age as a relational and cultural process inscribed in the continuity of the life cycle. Within this theoretical reading, normalization does not imply denying loss or vulnerability; rather, it allows age-related limits to be incorporated into an ongoing biography and addressed through care in the present.

Cluster 7, although also located within the area of risk, introduces a different dimension, probably influenced by its position in the factorial space in proximity to the positive pole of Factor 3, “Building one’s own life project.” The words problem, cognitive, retirement, theme, system, social, related, age, critical issue, and energy characterize this cluster, with problem emerging as the most central term. The label “The Problem of Old Age” reflects this lexical centrality. The centrality of the term “problem” may signal a shift to a further reflective level: here, considering old age as a problem may be interpreted as questioning possible simplifications regarding what it means to be old and to care for older people. In this configuration, old age and the care of older people may be understood as a complex problem situated at the intersection of multiple levels: the individual, the family, and society. From a theoretical perspective, this configuration can be connected to the idea that each of these levels is engaged in identifying resources and strategies that make old age no longer a condition that is merely endured and stereotyped, but rather a life phase whose meaning can—and must—be co-constructed and renegotiated (Wahl et al., 2021).

Among the illustrative variables, location (Rome vs. Bergamo) was the only variable showing a statistically significant association with cluster distribution at the context-unit level, whereas no statistically significant associations were observed for participants’ age or gender. Descriptively, context units from the Rome interviews occurred more frequently than expected in Clusters 5 (“Awareness”) and 6 (“Rethinking Services”) and less frequently than expected in Clusters 2 (“The Passing Years”) and 4 (“Denial”), with an opposite descriptive pattern observed for context units from the Bergamo interviews. The magnitude of the association was small (Cramér’s V = 0.19). Given that the chi-square analysis was conducted on context units nested within participants, and that this dependency was not explicitly modeled, this finding should be interpreted cautiously as an exploratory pattern in the corpus and not as evidence of a difference between participants from Rome and Bergamo. Despite the caution required in interpreting this finding for the reasons outlined above, some tentative interpretative considerations may be offered to provide a possible contextual reading of the observed pattern. The greater relative presence of Clusters 2 and 4 and the lower relative presence of Clusters 5 and 6 in the Bergamo portion of the corpus may suggest greater discursive salience of a representation of later life as a phase in which being active, productive, and useful constitutes an important condition for maintaining quality of life and for not “dropping out of the game.” At a further inferential level, this pattern may be compatible with a more individualistic and performance-oriented perspective, in which emphasis is placed on the older person’s ability to remain engaged and to maintain continuity with work as a source of identity and social value. Within this possible interpretation, limits, difficulties, or illness may receive comparatively less discursive space and may therefore appear less integrated within the representation of later life.

Conversely, the greater relative presence of Clusters 5 and 6 in the Rome portion of the corpus may suggest greater prominence of a socio-assistential and collective conception of old age, in which aging is represented in a more socially situated and potentially problematic manner, involving the need for change, shared responses, and a rethinking of service systems.

As a contextual hypothesis, these lexical differences may be considered in light of the distinct urban, socioeconomic, and institutional contexts associated with the cities in which participants reside and work. Rome is a large and internally heterogeneous metropolitan capital, whose territorial extension, demographic scale, and administrative organization across multiple municipal districts may increase the complexity of coordinating health, social, and community services. Bergamo, by contrast, is a medium-sized provincial city embedded in the wider northern Italian context. In such a context, work, productivity, and continued participation might constitute relatively salient cultural codes through which personal identity and social value are expressed.

These contextual explanations remain speculative, as the study did not directly assess socioeconomic conditions, organizational characteristics, service provision, professional culture, or individualistic and collectivistic orientations. They should therefore be understood as possible interpretative hypotheses rather than explanations of the observed association.

The absence of statistically significant associations with age and gender is also noteworthy but should be interpreted cautiously. It indicates only that no statistically significant associations were detected in the present analyses. This finding may be compatible with a relatively similar distribution of the identified configurations across age and gender groups within this corpus, but it does not demonstrate equivalence between these groups. The limited sample size, the uneven gender distribution, and the fact that multiple context units were contributed by the same participants may also limit the interpretation of potential demographic differences. Accordingly, and given the non-independence of context units within participants, these findings should not be interpreted as evidence that age or gender play no role in shaping representations of old age, nor as support for the conclusion that local, relational, organizational, or service contexts are more influential than individual demographic characteristics.

In conclusion, what may emerge from this research is that healthcare professionals, in their daily work, do not simply encounter old age as an external condition, but may also confront aspects of their own experience of adulthood and aging. In this sense, encounters with older adults may provide a space in which limitations, fears, possibilities, and less consciously considered aspects of adult life become salient. It is within this space, situated between proximity and distance, that the representations identified in the present study may take shape. From this perspective, working with such representations may involve not only technical but also symbolic and reflective dimensions. Creating spaces in which professionals can reflect on the meanings and experiences associated with old age may help them process the personal and professional resonances elicited by their encounters with older adults, potentially contributing to more reflective care practices. Such spaces may also support healthcare professionals in recognizing older adults in the complexity of their identities, beyond stereotypical or predominantly deficit-based representations. In this sense, the present findings may point towards the value of care environments that attend not only to the physical and practical needs of older adults, but also to their subjectivity and the relational dimensions of care. At the same time, supporting the reflective dimension of professional practice may contribute to greater awareness of the experiences and meanings that healthcare professionals themselves bring to their work.

### 4.1. Practical Implications

The findings suggest some potential practical implications for training, organizational practices, and service and policy development. At the training level, they suggest the potential value of integrating psychosocial and cultural dimensions of aging into the education and continuing professional development of healthcare professionals working with older adults, alongside clinical competencies. At the organizational level, the findings may support the development of multidisciplinary and community-based models of care that facilitate continuity, coordination, and interprofessional collaboration. At the service and policy level, they may inform approaches aimed at fostering the social participation of older adults, addressing institutional ageism, and supporting families involved in caregiving. These implications should nevertheless be interpreted cautiously. The present study was not designed to evaluate the effectiveness of specific interventions and therefore cannot establish whether such strategies would improve professionals’ well-being or the quality of care. Rather, they represent potential directions arising from the representations identified in the present study and warrant further empirical investigation.

As a methodological implication, future applications of ETM may benefit from dedicated training and hands-on activities to support researchers in the appropriate preparation, analysis, and interpretation of textual data.

### 4.2. Limitations

This study has several limitations that must be considered when interpreting the results. First, the sample consisted of a relatively small number of healthcare workers, recruited from only two Italian cities. While it offers a significant initial mapping of representations of old age, this limits the possibility of generalizing the results to the entire national or international contexts. Moreover, the non-probabilistic purposive and snowball sampling strategy may have introduced selection bias and limits the transferability of the findings to the broader population of healthcare professionals. Second, although ETM provides a systematic approach to identifying the symbolic structure of discourse, the final interpretation of factors and clusters requires a judgment-based step. Despite the use of comparison and triangulation procedures, this step may involve some degree of interpretative subjectivity. The relatively small sample size, the uneven distribution of age and gender, and the heterogeneity of professional roles, including very small occupational categories, also limited the possibility of conducting reliable subgroup analyses. Accordingly, the present study cannot establish whether the identified configurations are similarly distributed across different professional groups. Furthermore, the use of a single broad open-ended question allowed participants to draw on personal, social, cultural, and professional experiences. Although this was consistent with the exploratory aims of the study, the resulting narratives cannot be assumed to reflect exclusively professional representations of old age. The cross-sectional design also prevents conclusions regarding the direction of the relationship between professional experience and representations of old age.

An additional methodological limitation concerns the unit of analysis used in the chi-square analyses. Each context unit retained the age, gender, and location characteristics of the participant from whose interview it originated; however, multiple context units were generated from each participant. The 1409 retained context units were therefore not statistically independent, and their nesting within participants was not explicitly modeled. Moreover, participants contributed unequal numbers of context units, meaning that participants with longer or more extensively segmented interviews contributed more units to the analyses. Consequently, the effective amount of independent information is lower than the total number of context units, and the conventional chi-square test may underestimate the statistical uncertainty associated with the observed association. The association with location should therefore be interpreted as an exploratory corpus-level pattern rather than as confirmatory evidence of differences between individual participants from Rome and Bergamo. A further limitation concerns the fact that city of residence and city of professional practice coincided for all participants and were therefore operationalized as a single location variable. Consequently, the present analysis cannot determine whether the observed association reflects geographical context, professional practice context, or a combination of both. Future studies could address these limitations through larger and more diverse samples, a more differentiated assessment of professional and contextual factors, and participant-level or multilevel analytical approaches that explicitly account for the nested structure of textual data.

Lastly, although the use of a single open-ended question allowed participants to freely develop their narratives, the question encompassed several interconnected foci. Considering the findings and limitations of the present study, several areas for future research emerge. First, expanding the sample to include different Italian regions and care settings could help explore potential contextual and cultural variability in representations of old age. Future studies should also examine associations with participant characteristics using analytical approaches that explicitly account for the hierarchical structure of the data. Moreover, the use of visual prompts to help participants orient their narratives across different dimensions could be considered in future research.

## 5. Conclusions and Future Perspective

This study identified recurring configurations in the representations of old age emerging from the narratives of healthcare professionals working with older adults in two Italian cities. Within this specific corpus, old age emerged not simply through isolated judgments, but through recurrent patterns of meaning concerning proximity and distance, identification and protection, and the tension between caring for others and managing the emotional impact of care. Taken together, these configurations point to the complexity of later life as an object of care, in which personal, relational, cultural, and organizational dimensions may intersect.

The semantic space identified also suggests that, within this corpus, old age may be understood as a phenomenon extending beyond the individual and involving relationships with others, communities, institutions, and systems of care. The configurations concerning personal experience, relational dimensions, and the organization of services further suggest the potential relevance of considering aging not only as a clinical condition, but also as a socially and culturally embedded experience.

Despite the limitations discussed above, the present study provides an empirical basis for further examining the multiple and sometimes contrasting ways in which healthcare professionals may make sense of later life. Future studies could investigate whether similar configurations emerge across different professional groups, geographical areas, and care settings, using analytical approaches that explicitly account for the hierarchical structure of textual data. Longitudinal or intervention-based studies could also explore whether and under what conditions representations of old age may change over time or in response to specific reflective or organizational initiatives, without assuming in advance that such interventions would produce beneficial effects.

## Figures and Tables

**Table 1:** Sociodemographic Characteristics of Participants.

Characteristic	Total Sample *n* = 50	
	M	SD
Age	49.3	8.36
	**N**	**%**
Gender		
Female	37	74
Male	13	26
Location		
Bergamo	25	50
Rome	25	50
Professional role		
Psychologist	13	26
Physician	11	22
Healthcare assistant (OSS)	9	18
Nurse	4	8
Nurse manager	4	8
Professional educator	2	4
Neuropsychologist	2	4
Other	5	10

Note. “Other” included one social worker, one medical director, one physiotherapist, one speech and language therapist, and one psychomotor therapist.

**Table 2:** Correspondence analysis results.

Factor	Eigenvalues	Percentage	Cumulative Percentage
1	0.1809	23.52	23.52
2	0.1547	20.11	43.63
3	0.1278	16.61	60.24
4	0.1210	15.73	75.97
5	0.1079	14.03	90.00
6	0.0768	9.98	100

**Table 3:** Cluster placement in factorial space.

	FACTOR-1	FACTOR-2	FACTOR-3
CLUSTER_01	0.1101	−0.3642	−0.3039
CLUSTER_02	−0.6856	0.4197	−0.5959
CLUSTER_03	0.4073	0.5701	0.2789
CLUSTER_04	−0.4885	−0.3888	0.471
CLUSTER_05	0.3141	−0.3531	−0.1362
CLUSTER_06	0.4672	0.1565	−0.0438
CLUSTER_07	−0.2995	0.4375	0.4369

**Table 4:** Contingency table for variable location.

Cluster	Bergamo, Observed (Expected)	Rome, Observed (Expected)	Total
1. Witnessing the ageing process	153 (146.31)	64 (70.69)	217
2. The Passing Years	140 * (119.34)	37 * (57.66)	177
3. Challenging Old Age	127 (121.36)	53 (58.64)	180
4. Denial	196 * (174.63)	63 * (84.37)	259
5. Awareness	146 * (164.51)	98 * (79.49)	244
6. Rethinking Services	105 * (136.20)	97 * (65.80)	202
7. The Problem of Old Age	83 (87.65)	47 (42.35)	130
Total	950	459	1409

Note. * Marked cells identify the cluster-location combinations contributing most strongly to the significant overall association, based on descriptive examination of Pearson residuals.

## Data Availability

Anonymized data supporting the findings of this study are available from the authors upon reasonable request, due to privacy and confidentiality constraints.

## Supplementary Materials

The following supporting information can be downloaded at https://www.mdpi.com/article/10.3390/bs16091620/s1.
