# Supplementary material for: Exploring Representations of Old Age Emerging from the Narratives of Healthcare Professionals Working with Older Adults in Two Italian Cities
Source: Behav Sci (Basel). 2026 Sep 10;16(9):1620. doi: 10.3390/bs16091620 (PMC13603788; doi:10.3390/bs16091620)
Supplement: Supplementary file 1 [file behavsci-16-01620-s001.zip › behavsci-4483227-supplementary.pdf]

Consolidated criteria for reporting qualitative studies (COREQ): 32-item checklist

**No. Item**	**Guide questions/description**	**Reported on Page #**
**Domain 1: Research team and reflexivity**		
Personal Characteristics		
1. Inter viewer/facilitator	Which author/s conducted the inter view or focus group?	Two co-authors, A.G. and M.G., conducted the interviews. p. 4, Section 2.2 (Participants and procedure).
2. Credentials	What were the researcher's credentials? E.g. PhD, MD	Both interviewers held master's degrees in psychology. p. 4, Section 2.2.
3. Occupation	What was their occupation at the time of the study?	Both interviewers were psychologists. p. 4, Section 2.2.
4. Gender	Was the researcher male or female?	Both interviewers were female. p. 4, Section 2.2.
5. Experience and training	What experience or training did the researcher have?	Both interviewers had previous experience conducting qualitative interviews. p. 4, Section 2.2. The three judges had psychology backgrounds and ETM expertise; p. 5-6, Section 2.3.
Relationship with participants		
6. Relationship established	Was a relationship established prior to study commencement?	Neither interviewer had a prior relationship with participants. p. 4, Section 2.2.
7. Participant knowledge of the interviewer	What did the participants know about the researcher? e.g. personal goals, reasons for doing the research	Not reported beyond the researchers' stated purpose in the interview prompt. p. 4, Section 2.2.

8. Interviewer characteristics	What characteristics were reported about the inter viewer/facilitator? e.g. Bias, assumptions, reasons and interests in the research topic	Professional background, gender, qualifications, and interview experience are reported. Personal assumptions or interests were not reported. p. 4, Section 2.2.

**Domain 2: study design**		
Theoretical framework		
9. Methodological orientation and Theory	What methodological orientation was stated to underpin the study? e.g. grounded theory, discourse analysis, ethnography, phenomenology, content analysis	Exploratory qualitative design using Emotional Text Mining, grounded in a socio-constructivist perspective and a psychodynamic model of meaning-making. pp. 3–5, Introduction and Sections 1.1–2.2–2.3.
Participant selection		
10. Sampling	How were participants selected? e.g. purposive, convenience, consecutive, snowball	Purposive and snowball sampling. p. 4, Section 2.2.
11. Method of approach	How were participants approached? e.g. face-to-face, telephone, mail, email	Potential participants were contacted by telephone by a psychologist responsible for implementing the study protocol. p. 4, Section 2.2.
12. Sample size	How many participants were in the study?	Fifty participants (25 from Rome and 25 from Bergamo). pp. 1 and 4–5, Abstract, Section 2.2, and Table 1.
13. Non-participation	How many people refused to participate or dropped out? Reasons?	Of 53 professionals contacted, 3 declined; no reasons for refusal were recorded/reported. p. 5, Section 2.2.
Setting		
14. Setting of data collection	Where was the data collected? e.g. home, clinic, workplace	Individual, in-person interviews in a private setting. p. 4, Section 2.2.
15. Presence of non-participants	Was anyone else present besides the participants and researchers?	No non-participants were present. p. 4,

		Section 2.2.
16. Description of sample	What are the important characteristics of the sample? e.g. demographic data, date	Age, gender, location, and professional role are reported. pp. 4–5, Section 2.2 and Table 1.
Data collection		
17. Interview guide	Were questions, prompts, guides provided by the authors? Was it pilot tested?	The single open-ended question and the use of brief echo prompts are reported verbatim. Pilot testing was not reported. p. 4, Section 2.2.
18. Repeat interviews	Were repeat inter views carried out? If yes, how many?	No. Each participant completed one interview, with no follow-up interviews. p. 4, Section 2.2.
19. Audio/visual recording	Did the research use audio or visual recording to collect the data?	All interviews were audio-recorded and transcribed verbatim. p. 5, Section 2.3.
20. Field notes	Were field notes made during and/or after the inter view or focus group?	No field notes were taken. p. 5, Section 2.2.
21. Duration	What was the duration of the inter views or focus group?	Mean duration approximately 30 minutes; range 19–38 minutes. p. 5, Section 2.2.
22. Data saturation	Was data saturation discussed?	Data saturation was not used as a recruitment stopping criterion. Corpus adequacy was assessed using lexical indicators. P. 5, Sections 2.2.
23. Transcripts returned	Were transcripts returned to participants for comment and/or correction?	No. p. 5, Section 2.2.

**Domain 3: analysis and findings**		
Data analysis		
24. Number of data coders	How many data coders coded the data?	T-LAB generated the statistical outputs; three independent judges interpreted and labelled the factors and clusters. p. 6, Section 2.3.
25. Description of the coding tree	Did authors provide a description of the coding tree?	Not applicable in the conventional coding-tree sense. ETM used unsupervised lexical co-occurrence analysis, bisecting k-means clustering, and correspondence analysis; clusters and factors are reported in Sections 2.3 and 3.
26. Derivation of themes	Were themes identified in advance or derived from the data?	Derived from the data. ETM was bottom-up and unsupervised; judges independently interpreted statistical outputs before consensus. pp. 6–7, Section 2.3.
27. Software	What software, if applicable, was used to manage the data?	T-LAB version 10.2.6. p. 6, Section 2.3.
28. Participant checking	Did participants provide feedback on the findings?	No. Neither transcripts nor findings were returned to participants for comment or validation. p. 5, Section 2.2.
Reporting		

29. Quotations presented	Were participant quotations presented to illustrate the themes/findings? Was each quotation identified? e.g. participant number	Yes. Illustrative excerpts are presented for all seven clusters and identified by participant gender, location, and T-LAB score. pp. 12–11, Section 3.2.
30. Data and findings consistent	Was there consistency between the data presented and the findings?	Lexical units, chi-square values, cluster sizes, factorial positions, and illustrative excerpts are presented alongside the interpretations. Section 3.
31. Clarity of major themes	Were major themes clearly presented in the findings?	Yes. Seven clusters and the first three factorial dimensions are presented, with explained inertia and cluster proportions. pp. 8–11, Section 3 and Tables 2–3/Figures 1–2.
32. Clarity of minor themes	Is there a description of diverse cases or discussion of minor themes?	The seven clusters describe multiple and sometimes contradictory configurations. No separate formal negative-case analysis was conducted because the analysis was corpus-level and unsupervised. Sections 3–5.
